# Attempt to Quit Tobacco Smoking among Male Smokers in Vietnam and Predictive Factors: Findings from Global Adult Tobacco Survey (GATS)

**DOI:** 10.31557/APJCP.2021.22.7.2061

**Published:** 2021-07

**Authors:** Giang Bao Kim, Diep Bich Pham, Hai Thi Phan, Minh Dai Le, Minh Van Hoang

**Affiliations:** 1 *School of Preventive Medicine and Public Health, Hanoi Medical University, Hanoi, Vietnam. *; 2 *Viet Nam Tobacco Control Fund, MOH Viet Nam, Hanoi, Vietnam. *; 3 *Medical English Club, Hanoi Medical University, Hanoi, Vietnam. *; 4 *Hanoi University of Public Health (HUPH), Hanoi, Vietnam. *

**Keywords:** attempt to quit, tobacco smoking, tobacco cessation, Global Adult Tobacco Survey, Vietnam

## Abstract

**Background:**

Vietnam is among countries with highest prevalence of tobacco smoking, attempt to quit is an important indicator to monitor the effectiveness of tobacco control efforts. This paper aims to describe smoking quit attempt and examine its association with some individual characteristics among male smokers.

**Methods:**

Data from the Global Adult Tobacco Survey in Vietnam in 2015 was analyzed for a sample of 1,903 male smokers taking from the national representative sample of 8,996 adults aged 15 years and above.

**Results:**

Proportion of quit attempt during 12 months prior to the interview among male smoker was 37.1%. Attempt to quit smoking was significantly associated with age (OR=2.84 and 95% CI: 1.43-5.66 for those aged 55 years and older vs. those aged 24 years and younger), with knowledge of harmful effects on health (OR=1.97 and 95% CI: 1.45-2.66 for those who could list 6 to 7 diseases vs. those who could list 3 or less diseases), number of channels with anti smoking message (OR=1.72 and 95% CI: 1.21-2.45 for those who had exposure from 3 channels or more vs. those who did not expose any channels), number of years smoking (OR=0.59 and OR=0.40 for those with less than 15 years smoking vs. those with 25 to 34 years smoking and more than 35 years smoking, respectively).

**Conclusion:**

Intervention to improve knowledge of tobacco harmful effects, and access to multiple and modern antismoking communication channels would be effective to raise quit attempt among smokers. Research to promote effectiveness of quit advice by health staff should be paid more attention.

## Introduction

Globally, tobacco use is currently responsible for the deaths of about six million people across the world each year (World Health Organization, 2011). It is predicted that by 2030 smoking will kill more than 8 million people each year, and more than 80% of which will be in low and middle income countries if the current pattern persists (World Health Organization, 2008, 2011).

The Global Action Plan for the Prevention and Control of Non communicable Diseases during the period 2020 and 2030 identifies that 30% relative reduction of tobacco use in persons aged 15 years and above is among nine key targets to help prevent premature avoidable mortality from Non communicable diseases (World Health Organization, 2013). In order to assist tobacco control policies worldwide, WHO introduced a package of six evidence-based tobacco control demand reduction measures that are proven to reduce tobacco use in 2008, known as MPOWER package measures. Offer to quit smoking is an important component of tobacco control interventions at country level to reduce the demand for tobacco (World Health Organization, 2008). Health staff is expected to ask patients about tobacco use status and give advice to stop smoking if a patient reported using tobacco. Available evidences indicate that the brief advice to quit from a physician could improve cessation rates and is highly cost-effective (Stephen Babb et al., 2017). However, not all smokers got advice to quit smoking. In the United State of America, only 57.2% of adult smokers who had seen a health professional in the past year reported receiving advice to quit (Department of Health and Human Services, 2020).

Attempt to quit smoking varied across ages, socio-economc groups. It is more likely to happen among smokers with a higher level of nicotine dependence, more negative to smoking behavior (Eleni Vangeli et al., 2011; Hyland et al., 2016), who have higher level of motivation, who had higher level of confidence in quitting, who had previous quit attempt (Eleni Vangeli et al., 2011), who loose smoking friends over period of time (Bethany et al., 2016). Inversely, those who have greater enjoyment with smoking (Eleni Vangeli et al., 2011; Hitchman et al., 2014) and patients who are “disinterest in receiving advice” (Medawela et al., 2021) are less likely to make quit attempt. The relationship between age and education are not consistent between studies (Eleni Vangeli et al., 2011; Hitchman et al., 2014; Hyland et al., 2016)

Vietnam is among countries with highest prevalence of tobacco smoking. In 2010, the smoking prevalence among adults aged 15 years and above was 23.8%, and that was much higher among males (47.4%). Meanwhile, tobacco cessation was not well developed. According to the Global Adult Tobacco Survey (GATS) in 2010, only 29.7% of smokers got advice to quit by a health care provider in the a 12-month period (Ministry of Health of Viet Nam et al., 2010). In 2010, among male smokers, only 19.1% quitted smoking; 55.6% attempted to quit and 29.6% intended to quit smoking. The Law on Prevention and Control of Tobacco Harms in Vietnam in 2012 points out that tobacco cessation is an important measure to reduce smoking prevalence in Vietnam (National Assembly of Vietnam, 2012). Since then, Vietnam started to build and enhance cessation services and tobacco cessation counselling. In 2015, tobacco cessation counselling unit at a central hospital was established and several training activities have been conducted for 60 resource trainers and more than 600 health staffs at 63 provinces to prepare for the expansion of this service in other hospitals and health facilities (Vietnam Tobacco Control Fund, 2015). 

The Global Adult Tobacco Survey in 2015 (GATS 2015) in Vietnam is the second round of GATS in Vietnam after the first survey in 2010. The GATS aims to provide information to monitor tobacco control indicators in Vietnam according to MPOWER measures. In this second survey, tobacco cessation was also investigated. Updates on tobacco cessation indicators and its’ associated factors is valuable to plan for effective tobacco control activities. This paper used data from GATS 2015 in Vietnam to describe smoking quit attempts and association between quit attempt and socioeconomic and demographic factors among male smokers. 

## Materials and Methods

*Data source*

This paper used data from GATS 2015 in Vietnam. GATS is an international survey that was initiated by the Center for Diseases Control and the World Health Organization and financially supported by Bloomberg. In Vietnam, the survey was conducted by Ministry of Health, Hanoi Medical University, the General Statistics Office and with technical support from World Health Organization and Center for Diseases Control. The target population for this survey includes adults aged 15 years old or older in Vietnam. The sample of 9,513 households (4,725 in urban areas and 4,788 in rural areas) was calculated to obtain reliable estimates of the key variables of gender and urban or rural areas and covers 15% of total population. In each household, an adult aged 15 years old or older was randomly selected for the interview. The completed sample was 8,996 persons, yielding a response rate of 95.8%. Data collection was conducted by the General Statistics Office of Vietnam under technical supports of World Health Organization, Hanoi Medical University, Vietnam Steering Committee on Smoking and Health and Center for Disease Control and Prevention. An international standardized questionnaire was adapted through several steps of back translation procedures. The details descriptions was presented elsewhere (Ministry of Health of Viet Nam et al., 2016). Among 8,996 adults who completed the interviews, 1,972 persons reported as smokers in which 1,903 smokers were male. Thus, the sample used for this paper is 1,903 that equivalent to an weighted number of 15,213,471 male smokers according to Vietnamese population in 2015.

*Variables used*

Dependent variables were “attempt to quit smoking during the last 12 months”: smokers who reported at least one attempt to quit smoking during the last 12 months prior to the interviews. This can be any actions as the effort to stop smoking, such as joining direct advice session for cessation; using Nicotine replacement therapy; using regular chewing gum without any nicotine; using other prescription medications (for example, Bupropion, Varenicline), using traditional medicines; calling quit line or a smoking telephone support line; switching to smokeless tobacco; trying to quit without assistance.

Independent variables included (1) age groups; (2) education level, (3) occupation, (4) Wealth quintile based on household’s assets, (5) Residence (urban, rural); (6) Knowledge of diseases caused by tobacco smoking; (7) health staff ‘s advice to quit smoking when visiting health facilities during the last 12 months; (8) Number of communication channels for anti-smoking message during the 30 days prior to the interview; (9) Notice of health warning in cigarette package during the 30 days prior to the interview; (10) Number of years smoking; (11) Time after waking up for the first cigarettes (tobacco dependent levels).

*Data Analysis*

Descriptive statistics were performed to estimate frequencies and percentages for dependent and independent variables of interest. Prevalence of attempt to quit tobacco smoking was estimated for different groups based on individual characteristics. Logistic regression analyses were employed to examine the association between attempt to quit smoking and other factors among male smokers. Weight value were identified for each case in the database using following steps as described elsewhere (Global Adult Tobacco Survey Collaborative Group, 2020): “1) Determine the base weight to account for all steps of random selection that led to the sample of population members; 2) Adjust for nonresponse to compensate for sample imbalance due to differential success in sample recruitment; 3) Further adjust for incomplete frame coverage to adjust for imbalance due to a sampling frame that does not fully cover the population targeted for study; and 4. Further adjust to calibrate the final set of adjusted weights to the distribution of the population by characteristics that are highly correlated with the key study outcome measures (i.e., tobacco use behavior in GATS)” . Weights were used in all analyses, adjusted odds ratios (ORs) were estimated with 95% confidence intervals (CIs).

*Ethical considerations*

This survey complied with standard procedures that internationally used. It was approved by Ministry of Health of Vietnam, World Health Organization, Center for Disease Control and Prevention, and local authorities. Informed consent was gathered from respondent by the time of the interview. 

## Results

The proportion of male smokers at the age of 35 to 44 years old was highest (27%). The youngest group (less than 25 years old) was accounted for only 12.7%. More than half of male smokers had primary and secondary level of education (54.8%). Those had secondary and higher levels of education accounted for 15.7%. Self-employees accounted for 73.2%, following by employees of government and non-government organizations that accounted for 17.1%. Regarding knowledge of harmful effects on health of tobacco smoking, 48.1% male smokers could list only 3 or less diseases caused by tobacco. Of 68.8% male smokers lived in rural areas. 

During the last 12 months 11.9% got advice to quit tobacco smoking from health staff. During a 30 day-period prior to the interview, the proportions of male smokers did not receive the anti-smoking message from any media channels was 24.2%, received from 1-2 channels was 47.1%, from 3 channels and more was 28.7%. 

Among smokers, one third of them smoked less than 70 cigarettes per week (33.0%), one third of them smoked from 70 to less than 140 cigarettes (33.3%) per week and the other one third of them smoked 140 cigarettes per week (33.7%). 30.5% smokers had smoked for less than 15 years, 21.3% smoked tobacco for 35 years. More than half (53.6%) of smokers needed to smoke the first cigarette just 30 minutes after waking up. 

As shown in Table 3, the overall proportion of attempt to quit tobacco smoking among male smokers in Vietnam during 12 months prior to the interview was 37.1%. The proportion of attempt to quit smoking among male smokers at the oldest age group was highest at 40.3% and this was lowest at the youngest age group (34.9%) and those aged from 35 to 44 years (34.5%). The proportion of attempt to quit was highest among those male smokers with upper secondary education level (49%) and was lowest among those with lowest level of education (below primary: 29.6%). Those male smokers with more knowledge on harmful effects of tobacco on health had high rate of attempt to quit tobacco smoking (53.4% of those who could list 6 to 7 diseases caused by tobacco smoking compared to 32.3% of those who could list 3 or less diseases). Those who received advice to quit smoking from health staff had higher rate of attempt to quit compared to those who did not receive the advice (45.6% vs. 35.9%). There was a clear tendency that male smokers who received anti-smoking message during the last 30 days from more channels having higher rate of attempt to quit smoking (45.8%; 36.7% and 27.4% for groups had access to 3 channels or more, 1-2 channels; no channel at all, respectively). Those who had noticed health warning message on tobacco package during the last 30 days also had higher rate of attempt to quit as compared to those who did not (38.1% vs. 26.0%). Compared to those who smoked for less than 15 years, those smokers who had longer years of smoking (from 25 years and more) had a slightly lower rate of attempt to quit (35.8% and 35.5% vs. 39.1%). Those smokers who were more dependent to tobacco (shorter time after waking up having the first cigarette) had significantly lower rates of attempt to quit smoking: 32.73% among those smokers who belonged to the group “less than 30 minutes” compared to 39.5% of the group “from 30 to less than 60 minutes” and 47.09% of the group “60 minutes and longer”. 

The logistic regression models only found significant associations between attempt to quit smoking with age, knowledge of harmful effects on health, number of channels for anti smoking message, number of years smoking, and time after waking up for the first cigarette. Those who were 55 years and older 2.84 times more likely to attempt to quit smoking than those at the age group of 24 years and younger (95% CI: 1.43-5.66). Male smokers who could list 6 to 7 diseases caused by tobacco smoking 1.97 times more likely than those who could 3 or less diseases (OR=1.97; 95% CI: 1.45-2.66). Compared to those who did not receive anti-smoking message from any channels, the odds of attempt to quit was statistically significant higher among male smokers who had exposure to anti-smoking message from 3 channels or more (OR=1.72; 95% CI: 1.21-2.45). Compared to those smokers with less than 15 years smoking, the odds of attempt to quit smoking among smokers who had 25 to 34 years smoking and more than 35 years smoking were only 0.59 and 0.40 times, respectively. Compared to those who needed the first cigarette within 30 minutes after waking up, those who delayed smoking of the first cigarette after 60 minutes and longer were 1.65 times significantly more likely to attempt to quit (OR=1.65; 95% CI: 1.16-2.34); non daily smokers were 1.7 times more likely to attempt to quit (OR=1.70; 95% CI: 1.05-2.74). 

**Table 1:** Characteristics of the Study Participants (Weighted Numbers and Percentages)

Variables	Frequency		Weighted Percentage
	Un weighted	Weighted	
Age group			
<=24	118	1,798,362	12.4
25-34	308	3,198,218	22.9
35-44	432	3,839,393	27.5
45-54	468	2,690,562	19
>=55	457	2,644,988	18.2
Educational level			
Below primary school	332	2,669,299	17.6
Primary and basic secondary schools	1,035	8,333,293	54.8
Secondary school	292	2,392,554	15.7
Above secondary school	243	1,812,878	11.9
Occupation			
Government and non-government Employee	323	2,598,938	17.1
Self-employee	1,335	11,141,704	73.2
Unemployed (able and unable to work)	135	857,174	5.6
Student, Homemaker, Retired, others	110	615,655	4
Knowledge of harmful effects on health			
<= 3 diseases	918	7,324,903	48.1
4-5 diseases	557	4,547,023	29.9
6-7 diseases	428	3,341,545	22
Wealth quintiles			
Poorest	386	3,118,872	20.5
Second poorest	527	4,155,632	27.3
Averaged	290	2,482,949	16.3
Second richest	320	2,541,471	16.7
Richest	380	2,914,547	19.2
Residence			
Urban	874	4,742,270	31.2
Rural	1,029	10,471,201	68.8
Advice to quit by health staff			
No	1646	13,409,602	88.1
Yes	257	1,803,869	11.9
Number of channels for anti-smoking message			
No	442	3,688,708	24.2
1-2 channels	901	7,158,516	47.1
3 channels and more	560	4,366,247	28.7
Notice of health warning			
No	168	1,302,797	8.6
Yes	1,735	13,910,674	91.4
Total	1,903	15,208,024	100

**Table 2:** Smoking Status of Smokers

	Unweight	Weighted	Weighted Percentage
Number of cigarette per week			
Less than 70	599	5,023,669	33
From 70 to less than 140	642	5,069,759	33.3
140 and more	662	5,120,043	33.7
Number of years smoking			
Less than 15 years	408	4,633,289	30.5
15 -24 years	446	3,839,443	25.2
25 -34 years	499	3,505,730	23
35 years and more	550	3,235,009	21.3
Time after waking up for the first cigarette			
Less than 30 minutes	1,027	8,157,275	53.6
30-less than 60 minutes	315	2,470,226	16.2
60 minutes and longer	300	2,370,644	15.6
Non daily smoker	261	2,215,326	14.6
Total	1,903	15,213,471	100

**Table 3:** Percentage of Attempt to Quit among Male Smokers Aged 15 Years in Vietnam by Socioeconomic Status and Smoking Status

Variables	Weighted %	(95% CI)
Age group		
<=24	34.94	34.87-35.00
25-34	38.61	38.56-38.66
35-44	34.46	34.42-34.51
45-54	37.31	37.25-37.37
>=55	40.34	40.28-40.40
Educational level		
Below primary	29.61	29.56-29.67
Primary, basic secondary	38.79	38.75-38.82
Secondary	30.53	30.47-30.59
Above secondary	48.99	48.92-49.06
Occupation		
Employee	42.06	42.00-42.12
Self-employed	35.48	35.45-35.51
Unemployed	40.05	39.95-40.15
Other	40.9	40.78-41.03
Wealth quintiles		
Poorest	30.37	30.32-30.41
Second poorest	39.97	39.93-40.02
Averaged	39.42	39.36-39.47
Second richest	42.97	42.91-43.02
Richest	33.12	33.06-33.18
Knowledge of harmful effects on health		
<= 3 diseases	32.35	32.31-32.38
4-5 diseases	32.69	32.64-32.73
6-7 diseases	53.43	53.38-53.48
Residence		
Urban	36.94	36.90-36.99
Rural	37.14	37.11-37.17
Variables	Weighted %	(95% CI)
Advice to quit by health staff		
No	35.94	35.91-35.96
Yes	45.59	45.51-45.66
Number of channels		
None	27.42	27.38-27.47
1-2 channels	36.74	36.70-36.78
3 channels and more	45.79	45.75-45.84
Notice of health warning		
No	26.01	25.93-26.08
Yes	38.12	38.09-38.14
Number of cigrarettes per week		
Less than 70	39	38.96-39.05
From 70 to less than 140	38.93	38.89-38.98
From 140	33.36	33.32-33.4
Number of years smoking		
Less than 15 years	39.09	39.04-39.13
15 -24 years	37.14	37.09-37.19
25 -34 years *	35.79	35.74-35.84
From 35 years *	35.53	35.48-35.58
Time after waking up for the first cigarette		
Less than 30 minutes	32.73	32.70-32.77
30-less than 60 minutes*	39.5	39.44-39.56
60 minutes and longer*	47.09	47.03-47.15
Non daily smoker	39.67	39.60-39.73
Overall	37.08	37.05-37.1

**Table 4:** Logistic Regression Analyses of Association between Attempt to Quit Tobacco Smoking with Individual Factors among Male Smokers Aged 15 and Above, in Viet Nam in 2015

Variables	OR	95% CI
Age group		
<=24		
25-34	1.18	0.72-1.92
35-44	1.36	0.79-2.35
45-54*	1.89	1.02-3.51
>=55*	2.84	1.43-5.66
Educational level		
Below primary school		
Primary and basic secondary schools	1.20	0.86-1.67
Secondary school	0.80	0.51-1.25
Above secondary school	1.58	0.94-2.66
Occupation		
Employee		
Self-employee	0.99	0.69-1.42
Unemployed	1.12	0.61-2.07
Student, Homemaker, Retired, others	1.01	0.55-1.85
Wealth		
Poorest		
Second poorest	1.33	0.94-1.88
Averaged	1.18	0.79-1.77
Second richest	1.33	0.88-1.99
Richest	0.73	0.46-1.18
Knowledge of harmful effects on health		
<= 3 diseases		
4-5 diseases	0.95	0.72-1.25
6-7 diseases*	1.97	1.45-2.66
Residency		
Urban		
Rural	1.08	0.84-1.4
No. of channels for anti-smoking message		
None		
1-2 channels	1.31	0.95-1.8
3 channels and more*	1.72	1.21-2.45
Notice of health warning		
No		
Yes	1.51	0.98-2.33
Advice to quit by health staff		
No		
Yes	1.91	0.74-4.93
Number of cigarettes per week		
Less than 70		
From 70 to less than 140	1.06	0.76-1.48
140 and more	0.97	0.68-1.4
Number of years smoking		
Less than 15 years		
15 -24 years	0.82	0.54-1.26
25 -34 years *	0.59	0.36-0.97
35 years and more *	0.40	0.22-0.71
Variables	OR	95% CI
Time after waking up for the first cigarette		
Less than 30 minutes		
30-less than 60 minutes*	1.29	0.92-1.8
60 minutes and longer *	1.65	1.16-2.34
Non daily smokers	1.7	1.05-2.74

*, denotes a statistical significant result

## Discussion

In our study, the proportion of male smokers who attempted to quit tobacco smoking during 12 months prior to the interview was 37.1%. This is in accordance with results reported from Poland for the survey in 2010 (Dorota Kaleta et al., 2012). In China, the survey in found the rate of quit attempt is 25.3% (Lin Li et al., 2011) . We found that those who had higher level of knowledge on harmful effects of tobacco, who had noticed anti smoking message from more communication channels were significantly more likely to have quit attempts, with the odds ratios ranged from 1.72 (accessed to 3 channels for anti smoking messages or more vs. no channel); to 2.03 (could list 6-7 diseases caused by tobacco use vs. less than 4 diseases). This paper affirms that high level of knowledge of harmful effects of tobacco is predictor for quit attempt as reported in several papers. Study in Poland also indicates the positive relationship between high awareness of smoking health consequences with long-term quitting (Dorota Kaleta et al., 2012) . Survey in China (Lin Li et al., 2011) and in the Australia, Canada, the UK, and the USA (Hyland et al., 2016) among cigarette smokers confirm that negative opinion of smoking is predictor for attempt to quit among smokers. A report based on the Global Adult Tobacco Surveys in 17 countries reported that antismoking information in mass media channel, especially in multiple channels, significantly increased intent to quit smoking with the adjusted odds ratios ranging from 1.3 to 3.2 (Centers for Disease Control and Prevention, 2013). 

Although the proportion of smokers who were advised to quit smoking were significantly higher in 2015 as compared to that in 2010 (Ministry of Health of Viet Nam et al., 2010), the rate of quit attempt among male smokers in this survey of 2015 was lower than what were reported in the report of Global Adult Tobacco Survey in 2010 (55.3%) (Ministry of Health of Viet Nam et al., 2010). Our study could not support for the association between advice to quit by health staff and quit attempt among smokers. A study in Poland also supports this. The study found that 77% of smokers attempted to quit smoking on their own and only 11% of smokers who attempted to quit smoking because of physician’s investigation, and 41% of smokers prompted to quit by a personal health problems failed to see the link between their illness and tobacco smoking (Alicja et al., 2008). Roy by his review in 2017 indicates that smoking is a voluntary behavior that can be changed if smokers really want to or motivation appeared to promote them to stop (Roy, 2017). Thus, deeper researches about contents and effectiveness of quit advice by health staff should be conducted to propose appropriate solutions for more efficient advice by health staff. In addition, no improvement in the rate of quit attempt might be explained by the fact that access to anti smoking messages in 2015 did not increase as compared to that in 2010 (Ministry of Health of Viet Nam et al., 2010, 2016). Moreover, sources of anti smoking messages have not been changed in accordance with the trend in information sources preferred by the population, especially in younger generation. During the past few years, most of anti smoking messages have been presented in television and radio programs, while 58% of the population used less television than before (Vietnam Market Research, 2019). Furthermore, recently social media has become more and more popular, but anti smoking message has not been presented in these channels. According to statistics, the rate of using Internet in Vietnam increased from 30.7% in 2010 to 52% in 2016 (International Telecommunication Union (ITU) et al., 2019) and in 2018 among those using internet, 96% used YouTube, 95% used Facebook and more than 50% used Instagram, etc. (The Statistics Portal, 2019). Thus, in order to increase accessibility to anti smoking sources among population, communication programs through social media for anti smoking message are highly recommended (Vietnam Tobacco Control Fund, 2015). Smoking cessation program for young adults through Facebook are proved to be effective for quit attempt, reducing tobacco consumption and stop smoking (Danielle et al., 2015). Our findings support the previous studies indicating that the lower rate of quit attempt are observed among the smokers with higher dependent level (shorter time of the first cigarette after waking up) and with more years of smoking (Greenhalgh et al., 2016; Layoun et al., 2017). 

Our survey found statistical significant difference between age groups and that is in line with studies in other countries. Survey in China found that attempt to quit increased with increasing age (Lin Li et al., 2011) and that is in contrast with findings from the prospective cohort survey conducted in the Australia, Canada, the UK, and the USA among cigarette smokers that found that younger age were the factors predictive of making a quit attempt included intention to quit, making a quit attempt in the previous year (Hyland et al., 2016). We did not find any significant discrepancies with regards to quit attempt between occupations, and between wealth level groups. Possible reason for that could be no difference in accessibility and availability of information sources and cessation services between these groups in Vietnam because during the study period, anti-smoking messages were mostly communicated through common media channels and cessation services had not been developed yet. 

In conclusion, intervention to improve knowledge of tobacco harmful effects and antismoking communication by multiple and modern communication channels would be effective to raise quit attempt among smokers. Research to understand and promote effectiveness of quit advice by health staff on quit attempt and smoking cessation should be paid more attention. 

## Author Contribution Statement

All authors contributed to the manuscript. The first author is responsible for data analysis and making draft of the manuscript. All authors participated in adapting and testing the survey form that is original from and monitoring data collection process, commenting on data analysis and writing, completing the manuscript.
